# Components Related to Long-Term Effects in the Intra- and Interpersonal Domains: A Meta-Analysis of Universal School-Based Interventions

**DOI:** 10.1007/s10567-022-00406-3

**Published:** 2022-07-31

**Authors:** E. C. A. Mertens, M. Deković, M. van Londen, J. E. Spitzer, E. Reitz

**Affiliations:** 1 Clinical Child and Family Studies, Utrecht University, Heidelberglaan 1, 3584 CS Utrecht, Netherlands; 2 Developmental Psychology, Utrecht University, Heidelberglaan 1, 3584 CS Utrecht, Netherlands

**Keywords:** Components, Long-term effects, Universal school-based interventions, Intrapersonal domain, Interpersonal domain

## Abstract

The aims of the present meta-analysis were to (1) examine long-term effects of universal secondary school-based interventions on a broad range of competencies and problems and (2) analyze which intervention components were related to stronger or weaker intervention effects at follow-up. Fifty-four studies of controlled evaluations (283 effect sizes) reporting on 52 unique interventions were included. Long-term intervention effects were significant but small; effect sizes ranged from .08 to .23 in the intrapersonal domain (i.e., subjective psychological functioning) and from .10 to .19 in the interpersonal domain (i.e., social functioning). Intervention components were generally related to effects on specific outcomes. Some components (e.g., group discussions) were even related to both stronger and weaker effects depending on the assessed outcome. Moreover, components associated with long-term effects differed from those associated with short-term effects. Our findings underscore the importance of carefully selecting components to foster long-term development on specific outcomes.

PROSPERO registration number: CRD42019137981.

**Supplementary Information:**

The online version contains supplementary material available at 10.1007/s10567-022-00406-3.

## Introduction

By stimulating students’ psychosocial functioning, schools can play an important role in both helping students develop competencies and preventing them from developing problems in the intrapersonal domain (i.e., the ability to manage own feelings, emotions and attitudes towards the self), and the interpersonal domain (i.e., the ability to have positive relationships, understand social situations, and respond appropriately in social contexts) (Barber, 2005; Pellegrino & Hilton, 2012; Shek & Leung, 2016). To this end, many universal school-based interventions addressing both domains have been designed. Although these interventions aim to enhance students’ long-term development, little is known about their long-term effects. Knowledge about long-term intervention effects is pivotal as initial intervention effects may be sustained, fade-out, or increase over time (Gottfredson et al., 2015). Despite the importance of this information, most studies that evaluate interventions measure intervention effects only directly after the interventions have ended. In the meta-analysis of Durlak and colleagues (2011), which examined the effectiveness of social-emotional school-based interventions, only 33 (15%) of the included studies collected data at least 6 months after the intervention ended. To increase our understanding of long-term effects of universal school-based interventions, the current meta-analysis had two aims: (1) to evaluate the long-term effectiveness of universal school-based interventions in the intra- and interpersonal domains and (2) to identify which intervention components were associated with stronger or weaker long-term intervention effects.

### Long-Term Effects of Universal School-Based Interventions

The few studies that did examine long-term effects of universal school-based interventions showed that these effects appear to be modest and seem to fade-out over time. For instance, Dray et al. (2017) found in their systematic review of universal school-based resilience-focused interventions that the positive intervention effects on anxiety and depressive symptoms extinguished over time. Decreasing or no long-term intervention effects are also reported by Mackenzie and Williams (2018) in their review of universal school-based interventions promoting mental and emotional wellbeing in the UK as well as by Sklad et al. (2012) in their meta-analysis of school-based social, emotional, and behavioral interventions. Taylor et al. (2017) conducted a meta-analysis of long-term intervention effects of Social Emotional Learning (SEL) interventions, and while they found positive long-term intervention effects, these effects were generally smaller than the short-term effects of the same interventions on SEL skills, attitudes, positive social behavior, conduct problems, and emotional distress (Durlak et al., 2011).

Follow-up intervention effects seem especially modest for adolescents. Both Dray et al. (2017) and Taylor et al. (2017) found fewer and weaker long-term intervention effects for adolescents than for children on indicators of general wellbeing as well as indicators of a disadvantageous development, such as anxiety and psychological distress (e.g., *Cohen’s d* = 0.27 for 5–10 years old versus *Cohen’s d* = 0.12 for 11–13 years old on general wellbeing; Taylor et al., 2017). Moreover, Taylor et al. (2017) showed that only a small proportion of the included interventions (11 of the 82, or 13%) targeted adolescents (14–18 years old). While Dray et al. (2017) included more interventions targeting adolescents (38 of the 57, or 67%), these studies focused only on problematic outcomes such as depression and anxiety. This lack of a broad focus on adolescent development is remarkable, as adolescence is an important developmental phase in which youth start to consolidate their own identity and encounter increasing opportunities for social interactions (Barber, 2005). To gain insight in the extent to which universal school-based interventions can cultivate adolescents’ long-term psychosocial development, the current meta-analysis focused specifically on interventions targeting adolescents. Hence, we included only secondary school-based interventions, rather than interventions targeting both children and adolescents as in previous meta-analyses and systematic reviews (e.g., Dray et al., 2017; Taylor et al., 2017).

Furthermore, in contrast to previous meta-analyses, we made a distinction between outcomes in the intrapersonal domain and outcomes in the interpersonal domain. These two domains are related, but distinct. How people view themselves can affect the way they approach social interactions, and vice versa (Finkel & Vohs, 2006). However, where the intrapersonal domain reflects one’s subjective psychological functioning, the interpersonal domain reflects one’s social functioning (Dufner et al., 2019). We examined a broad range of outcomes regarding competencies and problems in both domains. Competencies reflect skills and capacities that may contribute to adolescents’ healthy psychosocial development, whereas problems are difficulties and vulnerabilities that can heighten adolescents’ chance of developing psychopathology (Van Orden et al., 2005). In addition, we tested the extent to which the duration of the follow-up period affected intervention effects. We hypothesized that school-based interventions establish small positive effects at follow-up and that these long-term intervention effects are moderated by the duration of follow-up period, with a longer follow-up period relating to a decline in intervention effects (i.e., fade-out; Dray et al., 2017; Mackenzie & Williams, 2018; Sklad et al., 2012; Taylor et al., 2017).

### Intervention Components and Follow-Up Effects

Besides examining the effectiveness of universal secondary school-based interventions at follow-up, the present meta-analysis aimed to extend previous research by examining which intervention components were related to stronger or weaker intervention effects at follow-up. Previous meta-analyses examining school-based interventions examined few, if any, components and analyzed how these relate to intervention effects immediately after the intervention (e.g., Mertens et al., 2020; Sheridan et al., 2019). To increase our understanding of long-term intervention effects, we must unravel which intervention components are related to long-term effects. Therefore, we studied a broad range of components and how they relate to intervention effects at follow-up. Studying different types of components provides an elaborate overview of which components are interesting to study further when aiming to cultivate a specific outcome or domain. Based on this knowledge, we can optimize interventions through the addition of effective components and the elimination of components related to weaker long-term effects (Sheridan et al., 2019). In addition, this knowledge will enable schools to select and implement interventions according to their use of evidence-based components (Nocentini et al., 2015). As schools have limited time and resources to invest in implementing interventions, this guidance is of great practical value.

We focused on three types of intervention components: Content components, instructional components, and structural components. *Content components* represent specific skills that are taught to enhance positive outcomes, i.e., “what they learn”, such as emotion regulation and problem solving (Boustani et al., 2015). *Instructional components* represent techniques and information delivery methods applied by the facilitator of the intervention, i.e., “how they learn it”, such as modeling and multimedia use (Boustani et al., 2015). *Structural components* represent the structure of the intervention that may affect intervention effects, i.e., “how the intervention is set up”, such as parental involvement and the number of sessions (Lee et al., 2014).

All three types of components haven been related to short-term intervention effects. Regarding *content components* related to short-term intervention effects, Gaffney et al. (2021) found in their meta-analysis of anti-bullying interventions that the component of ‘teaching social-emotional skills’ was related to weaker effects on bullying and victimization. The meta-analysis of universal school-based interventions of Mertens et al. (2020) looked at more specific components of teaching social-emotional skills and found that focusing on emotion regulation, assertiveness, and/or cognitive coping were related to weaker effects. On the other hand, focusing on insight building and problem solving related to stronger effects. Concerning *instructional components* and short-term effects, using the component of ‘an active learning approach’ (i.e., methods in which students interact with others and perform tasks) is consistently related to stronger intervention effects. For instance, practicing the new skills learned during the intervention as well as modeling of desired behaviors have been associated with stronger effects in, respectively, universal school-based interventions (Mertens et al., 2020) and family-school interventions (Sheridan et al., 2019). Regarding *structural components* and short-term effects, longer and more extensive school-based interventions seem, in general, to be associated with stronger intervention effects. Implementing more sessions and involving more people in the intervention (e.g., whole-school approach, parental involvement) has been related to stronger effects on students’ mental health (Mertens et al., 2020; Sheridan et al., 2019), school climate (Mertens et al., 2020), and bullying (Gaffney et al., 2021; Mertens et al., 2020; Sheridan et al., 2019).

Although these meta-analyses provide a useful overview of intervention components related to short-term effects, little is known about components related to long-term intervention effects. It is important to study associations between components and short-term as well as long-term effects, as these associations may differ. For instance, a component unrelated to short-term effects may be related to long-term effects, thus representing a ‘sleeper effect’ in which intervention effects emerge or increase over time after the conclusion of the intervention (Bell et al., 2013; Van Aar et al., 2017). Therefore, the second aim of the current meta-analysis was to identify components related to stronger and weaker intervention effects at follow-up. Studying how components relate to both stronger and weaker effects enabled us to examine ‘what might work’ as well as ‘what might *not* work’. Given that previous research has not yet examined relations between components and long-term effects of universal school-based interventions, this aim was exploratory.

## Method

The current meta-analysis was part of a larger project examining associations between intervention components and intervention effects (PROSPERO registration number: CRD42019137981). Therefore, our meta-analysis applied the same method as the meta-analysis of Mertens et al. (2020).

### Inclusion and Exclusion Criteria

We aimed to include studies that evaluated universal secondary school-based interventions that intended to stimulate competencies and/or prevent the development of problems in students’ intra- and interpersonal domains. Universal secondary school-based interventions were defined as interventions implemented during regular school hours that targeted all students (Mychailyszyn et al., 2012; Peters et al., 2009). The intrapersonal domain was defined as the ability to manage one’s own feelings, emotions, and attitudes about the self (Barber, 2005). The interpersonal domain was defined as the ability to build and maintain positive relationships with others; to understand social situations, roles, and norms; and to respond appropriately in social contexts (Pellegrino & Hilton, 2012; Shek & Leung, 2016). Students’ abilities in both domains can facilitate personal functioning represented by competencies (e.g., resilience, self-regulation, social competence) or obstruct personal functioning represented by experienced problems (e.g., internalizing behavior, aggression, bullying; Dufner et al., 2019; Finkel & Vohs, 2006). Definitions of the two general domains and the subdomains are provided in the online Appendix A.

Inclusion criteria were: (1) the intervention was implemented in a regular school setting (i.e., schools providing special needs education were excluded), (2) the intervention took place during regular school hours in a group setting, (3) the intervention aimed to improve competencies and/or prevent problems in the intra- and/or interpersonal domain (i.e., interventions primarily aiming to improve students’ physical health (e.g., prevention of substance use, nutrition, pregnancy, STDs) or prevent a specific disorder (e.g., depression) were excluded.), (4) the intervention was universal, so targeting all students at the school, (5) the participants were in middle school or high school (Grades 6–12), (6) the study included a control group, (7) the study included a quantitative baseline and follow-up measurement of (subdomains of) the intrapersonal domain and/or interpersonal domain, (8) sufficient information concerning baseline and follow-up measurements was reported, or obtained after contact with the author, so that effect sizes could be calculated at follow-up, corrected for baseline differences, (9) the study was written in English, and (10) the study was published as article, book, or book chapter. We did not include unpublished studies as their inclusion does not reduce the possible impact of publication bias and can even be counterproductive, due to selection bias (Ferguson & Brannick, 2012).

### Literature Search

**Fig. 1:**
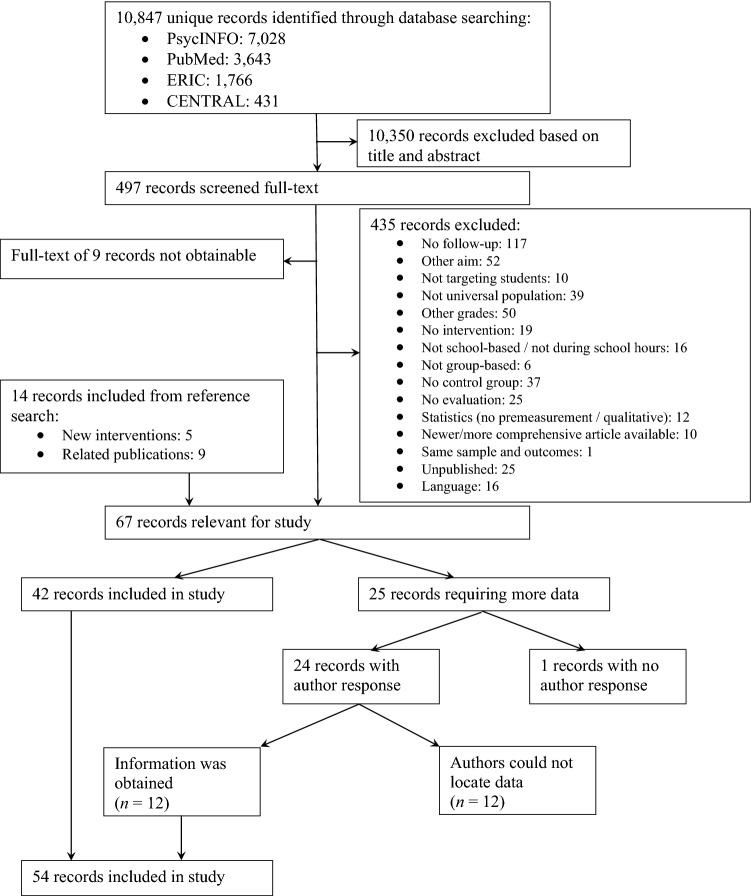
Flow chart

We searched four databases (i.e., PsycINFO, PubMed, ERIC, and CENTRAL) to cover psychological, medical, and education literature without a restriction on time period. We searched for randomized controlled trials and quasi-randomized controlled trials as both study designs feature a comparison between an intervention and a control condition (though the extent of random allocation to the condition differs between the two designs). Search terms to obtain school literature (e.g., school, class), interventions (e.g., prevention, intervention), adolescents (e.g., adolescent, youth), and intra- and interpersonal outcomes (e.g., self-esteem, social competence) were used. Given that this search results in an extremely high number of studies, restrictions to the search were added. These restrictions aimed to avoid interventions that targeted other populations (e.g., preschool, clinical) or outcomes (e.g., substance use, lifestyle) than those targeted in this study (see online Appendix B for the complete search string). This search (January 2021) resulted in 7,028 studies in PsycINFO, 3643 studies in PubMed, 1766 studies in ERIC, and 431 studies in CENTRAL. After duplicates were removed, 10,847 unique studies remained. Additionally, we searched the reference lists of included studies and relevant reviews and meta-analyses, resulting in an additional 14 studies. As a first step, all identified studies were screened by the first author based on title and abstract, which led to the exclusion of 10,350 studies (95%). The author then screened the full-texts of the remaining 497 studies, resulting in the exclusion of another 444 studies (89%). See Fig. 1 for the flow diagram.

One of the co-authors independently screened a random selection of studies to assess reliability of the two screening phases. To this end, 11% (1175 studies) of all identified studies and 10% (48 studies) of the studies remaining after the first screening were checked for relevance for inclusion in the present meta-analysis. Reliability was corrected for prevalence bias due to the high rate of exclusion over inclusion (Hallgren, 2012). Reliability for both screening phases was good (title/abstract: 96% agreement, prevalence corrected κ = 0.96; full-text screening: 98% agreement, prevalence corrected κ = 0.96). Disagreements regarding inclusion of studies were solved through discussion between the two researchers.

### Data Extraction

Studies were coded for information concerning the study (e.g., year of publication, country where study was conducted), sample (e.g., age, gender distribution), design and method (e.g., randomization, attrition analyses), intervention (e.g., intervention provider, aim of intervention), effect size data (e.g., outcome category), and intervention components (e.g., problem solving, practice, parental involvement). The intervention components were primarily based on the meta-analysis by Boustani and colleagues (2015) who, in turn, based their components on the PracticeWise Clinical Coding System (PracticeWise, 2009). An overview of all components and their definitions is presented in the online Appendix C. Sources cited in the study and other freely available materials, such as descriptions from the developer or websites, were retrieved for coding the components (Boustani et al., 2015; Kaminski et al., 2008). In cases where insufficient data were reported for calculating the effect size, the first author was contacted. When this author had not responded after a reminder, the second or last author was contacted and, if necessary, reminded. If the required data could not be obtained after this, the study was excluded from the meta-analysis (See Fig. 1 for the flow diagram). Key characteristics of the included interventions are provided in the online Appendix D.

Of the included studies, 30 studies (28%) were coded independently by a second coder for reliability. The inter-rater reliability was moderate to excellent with an average intra-class-correlation of 0.97 (*SD* = 0.05), ranging from 0.88 to 1.00, for continuous variables, and an average Cohen’s kappa of 0.82 (*SD* = 0.11), ranging from 0.60 to 1.00, for categorical variables. An exception was coding of the component ‘Insight building’ which had a slightly lower reliability (Cohen’s kappa of 0.52). Disagreements between the two coders were discussed and solved unanimously.

### Calculation and Analyses of Effect Sizes

Effect sizes reflected standardized mean differences between the intervention and control condition (Cohen’s *d*), following the procedures of Lipsey and Wilson (2001). Effect sizes were calculated for the first follow-up measurement after the post-measurement, unless the post-measurement was conducted more than 6 months after the end of the intervention. In those cases, the post-measurement was treated as a follow-up (Gottfredson et al., 2015). Effect sizes were adjusted for baseline differences and for Hedges’ (1983) small sample correction. The effect sizes were screened on outliers and winsorized by replacing outliers with the lower or upper value of two standard deviations from the mean (Lipsey & Wilson, 2001).

### Publication Bias

Given that studies with nonsignificant or negative results are less likely to be published than studies with significant or positive results, we assessed publication bias using a funnel plot. Effect sizes of studies are assumed to be symmetrically distributed around a true effect size represented by a funnel with at the top more precise effect sizes and at the bottom less precise effect sizes. An asymmetrical distribution of effect sizes can be an indication of publication bias (Light & Pillemer, 1984). The symmetry of a funnel plot can be tested with Egger’s regression test (Egger et al., 1997). In case of an asymmetrical funnel plot, indicated by Egger’s regression test, the effect for possible publication bias can be adjusted with the trim-and-fill analysis (Duval & Tweedie, 2000a, 2000b). Studies that fall outside the symmetric part of the funnel plot are estimated and trimmed. Subsequently, the true center of the trimmed funnel plot is estimated and the trimmed studies and their missing counterparts are replaced in the funnel. The corrected mean of this filled funnel plot can be estimated providing an effect size adjusted for possible publication bias. However, these tests assume independence of effect sizes which is not the case in multilevel meta-analyses. We took dependency among effect sizes into account by including the variance of the effect sizes as a moderator in Egger’s regression test. This approach was not possible in the trim-and-fill analysis, since no moderator can be added. To have some indication of potentially missing effect sizes, the trim-and-fill method was used for sensitivity analyses.

### Analyses

For each measure reported in the studies that fell within the intra- or the interpersonal domain, we calculated an effect size. Furthermore, we examined the time span between the end of the intervention and the follow-up measurement as a moderator of intervention effects. We took the clustering of effect sizes within a study into account by using multilevel meta-analytical models with three levels. The first level models sampling variance around each effect size. The second level models variance between effect sizes within studies. The third level models variance between studies (Assink & Wibbelink, 2016; Van den Noortgate et al., 2013).

Given that we were interested in the interventions’ effectiveness compared to the control condition, we used interventions as the unit of analysis. Hence, if a publication examined two interventions, both interventions were included and analyzed separately. If multiple publications examined the same intervention, though evaluated in different samples, the effect sizes of these publications were analyzed within the same intervention cluster. If multiple publications examined the same intervention within the same sample, we coded the most comprehensive publication. The less comprehensive publication was checked for additional information.

The multilevel analyses were conducted in R using the metaphor package (Viechtbauer, 2010). We analyzed the overall effectiveness at follow-up of universal school-based interventions on students’ intra- and interpersonal domains in separate models. To assess the extent to which these effect sizes at follow-up reflected intervention effects rather than methodological influences or biases, we examined methodological rigor (Lipsey & Wilson, 2001) based on the Cochrane Risk of Bias 2.0 tool for Cluster Randomized Trials (Higgins et al., 2016). We analyzed randomization (random vs. quasi-random assignment), completeness of outcome data (percentage of drop-out), and type of comparison group (passive: No intervention/waitlist vs. active: Care as usual/other intervention) as covariates. Characteristics of methodological rigor that were significantly related to the overall effect sizes at follow-up were included in further analyses as covariates.

Associations between components and intervention effects at follow-up were examined through moderation analyses. Moderation analyses were only conducted when both levels of the moderator (i.e., component present or not) contained at least three effect sizes (Crocetti, 2016). We report on significant effects (*p* < 0.05) as well as effects that trend towards significance (*p* < 0.10) given the low power of some of our analyses. In addition, these findings contribute to the hypotheses generating character of the meta-analysis and help illustrate the generalizability of moderation effects to other outcomes.

## Results

### Descriptive Characteristics

In total, 54 studies were included that reported on 52 unique interventions, from which we extracted 283 effect sizes. Of these intervention effects, 128 were in the intrapersonal domain and 155 were in the interpersonal domain. Six effect sizes from two studies (Bonell et al., 2018; Kaveh et al., 2014) were extreme outliers (Cohen’s *d* = 1.38–3.69) and believed to be unrepresentatively high. We therefore winsorized these six effect sizes.

The included studies were published between 1988 and 2021 (median year of publication: 2015). They were conducted in Europe (*k* = 26), the USA (*k* = 13), Australia (*k* = 6), Asia (*k* = 5), Africa (*k* = 2), and Canada (*k* = 2). Studies mostly allocated participants randomly to the conditions (*k* = 37) and slightly more often used a passive control group (*k* = 32) than an active control group (*k* = 22). On average, the follow-up measurement occurred 27.70 weeks (*SD* = 20.80) after the end of the intervention. The follow-up periods ranged from 2 to 109 weeks with 4 studies (7.4%) having a follow-up period of less than 3 months and 42 studies (78%) having a follow-up period of 24 weeks or longer.

In total, 51,017 participants participated in the included studies with an average age of 13.65 years (*SD* = 1.43) at the start of the intervention. Roughly half of these participants were boys (46%). Of the studies that reported on participants’ ethnic backgrounds (*k* = 31), 20 (37%) included participants mainly from an ethnic majority background, 8 (15%) included participants mostly from an ethnic minority background, and 3 (6%) included participants from a mix of majority and minority ethnic backgrounds. On average, participants’ drop-out rate in the studies was 17% (*SD* = 14.20). The interventions consisted of 11 sessions (*SD* = 7.32) on average, with an average timespan of 20.85 weeks (*SD* = 28.36). Teachers conducted the interventions about half the time (*k* = 24), while professionals conducted the other half (*k* = 30). Additionally, peers were involved in the implementation of 3 interventions.

### Overall Effect Sizes at Follow-Up

In the intrapersonal domain, interventions had a small positive effect at follow-up (*d* = 0.19; see Table 1). Intervention effects on self-esteem and general wellbeing were slightly stronger than on self-regulation. The effect size of internalizing behavior was significant but negligible (*d* = 0.08). No significant intervention effect was found on resilience. Also in the interpersonal domain, interventions had a small positive effect at follow-up (*d* = 0.16). The strongest effect was found on aggression. Intervention effects on sexual health and social competence were significant and comparable in magnitude. The intervention effect on bullying was also comparable in magnitude, though not significant.

**Table 1:** Effectiveness of interventions in the intra- and interpersonal domains

Domains	Number of effect sizes (*k*)	Effect sizes	95% CI
Intrapersonal	128	.19	.08; .30
Resilience	10	.14	− .12; .39
Self-esteem	32	.23	.08; .37
Self-regulation	18	.13	.05; .20
Wellbeing	33	.22	.02; .43
Internalizing problems	34	.08	.01; .14
Interpersonal	155	.16	.09; .23
Sexual health	46	.10	.03; .16
Social competence	22	.11	.03; .19
Aggression	58	.19	.06; .32
Bullying	21	.14	− .04; .32

The duration of the follow-up period did not affect the magnitude of intervention effects, other than for intervention effects on general wellbeing and social competence. A longer time span between the intervention and the follow-up was related to stronger intervention effects on general wellbeing [*B* = 0.25, *p* = 0.009, 95% CI (0.07; 0.43)]. In contrast, a longer time span between the intervention and follow-up was related to weaker intervention effects on social competence [*B* = − 0.09, *p* = 0.006, 95% CI (− 0.15; − 0.03)].

Analyses examining methodological rigor showed that effect sizes at follow-up concerning the intrapersonal domain, the interpersonal domain, and the subdomains did not depend on methodological considerations, i.e., the randomization of participants, rate of drop-out, or choice of comparison group.

### Publication Bias

**Fig. 2:**
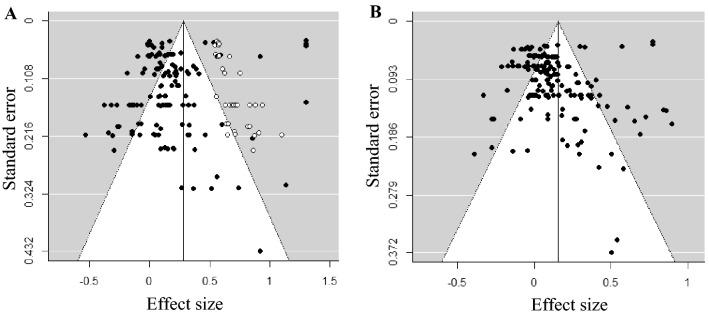
(Trimmed and Filled) funnel plots for (**A**) the intrapersonal domain with closed dots indicating observed effect sizes and open dots the filled effect sizes, and (**B**) the interpersonal domain

The distribution of effect sizes in the intrapersonal domain appeared to be asymmetrical (Egger’s regression test *z* = − 4.02, *p* < 0.001; see Fig. 2a), showing that there was a risk of publication bias. The trim-and-fill analysis indicated that 35 effect sizes were missing on the top right, meaning that large scale studies with larger effect sizes were missing. The adjusted effect size was 0.28 [95% CI (0.23;0.34)]. This sensitivity analysis, in which we could not correct for dependency among effect sizes, indicated that the original effect sizes provided a conservative estimate of the overall intervention effect. We therefore conducted further analyses based on the original effect sizes. Regarding the interpersonal domain, the distribution of effect sizes appeared to be symmetrical (Egger’s regression test *z* = 0.59, *p* = 0.558; see Fig. 2b), indicating that there was a low risk of publication bias.

### Intervention Components Related to Intervention Effects at Follow-Up

#### Descriptive Analyses

**Fig. 3:**
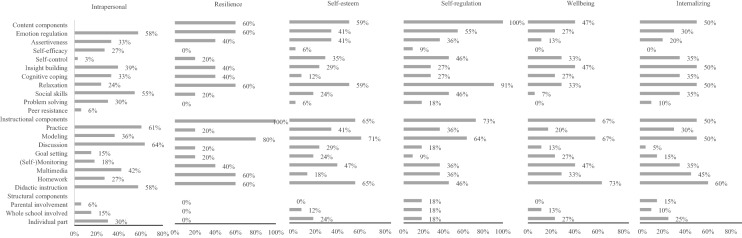
Frequencies of components used in interventions stimulating the intrapersonal domain and specific competencies and problems

**Fig. 4:**
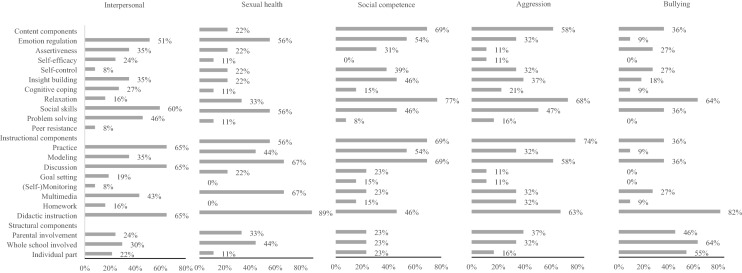
Frequencies of components used in interventions stimulating the interpersonal domain and specific competencies and problems

Content and instructional components that are commonly used in interventions to stimulate competencies and prevent problems in the intrapersonal domain (see Fig. 3) are generally the same components as those used in interventions addressing the interpersonal domain (see Fig. 4). Commonly used components are teaching students emotion regulation and social skills (content components), practicing skills during the intervention, facilitating discussions, and providing didactic instructions (instructional components). Interventions differ, though, in their commonly used structural components. Interventions addressing the intrapersonal domain often include an individual aspect, whereas interventions addressing the interpersonal domain often involve parents and/or the whole school in the intervention.

#### Intrapersonal Domain

None of the components was significantly associated with intervention effects at follow-up on the intrapersonal domain in general (see Table 2). Regarding the subdomains, insight building and multimedia use were related to stronger intervention effects on self-regulation, whereas teaching students problem solving was related to weaker effects on this outcome. Having group discussions was associated with stronger effects on internalizing behavior and involving the whole school in the intervention was related to stronger effects on students’ general wellbeing.

**Table 2:** Effect sizes of interventions with and without components stimulating competencies and preventing problems in the intrapersonal domain

Component		Intrapersonal			Resilience			Self-esteem			Self-regulation			Wellbeing			Internalizing		
		#ES	ES	*B*	#ES	ES	*B*	#ES	ES	*B*	#ES	ES	*B*	#ES	ES	*B*	#ES	ES	*B*
Content components																			
Emotion regulation	Yes	86	.18	− .03	8	–	–	17	.16	− .17	18	–	–	21	.33	.23	21	.08	.00
	No	42	.21		2			15	.33		0			12	.10		13	.07	
Assertiveness	Yes	52	.21	.04	8	–	–	11	.25	.03	7	.12	− .02	9	.14	− .11	15	.05	− .04
	No	76	.18		2			21	.22		11	.13		24	.25		19	.09	
Self-efficacy	Yes	45	.26	.10	7	.28	.39^†^	17	.27	.07	5	.18	.09	5	.06	− .18	10	.11	.05
	No	83	.16		3	− .11		15	.20		13	.09		28	.25		24	.06	
Self-control	Yes	125	.23	.04	0	–	–	2	–	–	1	–	–	0	–	–	0	–	–
	No	3	.19		10			30			17			33			34		
Insight building	Yes	48	.18	− .02	6	.26	.27	8	.26	.05	11	.19	.16*	15	.25	.05	9	.12	.06
	No	80	.20		4	− .01		24	.21		7	.03		18	.20		25	.06	
Cognitive coping	Yes	57	.14	− .07	2	–	–	9	.21	− .03	4	.05	− .09	18	.17	− .10	22	.08	− .00
	No	71	.21		8			23	.24		14	.14		15	.27		12	.08	
Relaxation	Yes	28	.13	− .08	2	–	–	4	.15	− .09	3	.12	− .01	5	.10	− .16	13	.09	.03
	No	100	.21		8			28	.24		15	.13		28	.26		21	.07	
Social skills	Yes	80	.22	.06	8	–	–	16	.26	.07	17	–	–	18	.38	.26	20	.08	.00
	No	48	.15		2			16	.19		1			15	.12		14	.08	
Problem solving	Yes	31	.13	− .08	6	.26	.27	7	.30	.10	5	.03	− .14*	2	–	–	11	.03	− .08
	No	97	.21		4	− .01		25	.21		13	.17		31			23	.11	
Peer resistance	Yes	5	.01	− .20	0	–	–	1	–	–	2	–	–	0	–	–	2	–	–
	No	123	.21		10			31			16			33			32		
Instructional components																			
Practice	Yes	88	.21	.05	10	–	–	18	.27	.11	15	.16	.13^†^	23	.17	− .15	21	.10	.05
	No	40	.16		0			14	.06		3	.03		10	.32		13	.05	
Modeling	Yes	37	.15	− .06	6	.26	.27	11	.13	− .15	4	.16	.04	6	.23	.01	10	.11	.04
	No	91	.21		4	− .01		21	.29		14	.11		27	.22		24	.07	
Discussion	Yes	95	.21	.06	9	–	–	27	.23	.01	14	.16	.14^†^	23	.17	− .14	21	.13	.13*
	No	33	.15		1			5	.22		4	.03		10	.31		13	− .00	
Goal setting	Yes	25	.21	.03	6	.26	.27	8	.23	.00	2	–	–	8	.24	.02	1	–	–
	No	103	.19		4	− .01		24	.23		16			25	.22		33		
(Self-)monitoring	Yes	31	.24	.06	1	–	–	6	.21	− .02	2	–	–	12	.21	− .01	9	.14	.08
	No	97	.18		9			26	.23		16			21	.22		25	.06	
Multimedia	Yes	56	.22	.05	2	–	–	17	.32	.19	10	.19	.15*	17	.16	− .11	11	.14	.11^†^
	No	72	.17		8	.11	.00	15	.13		8	.04		16	.27		23	.04	
Homework	Yes	38	.10	− .12	3	.10		4	.19	− .05	5	.04	− .12	8	.08	− .20	17	.04	− .07
	No	90	.23		7			28	.24		13	.16		25	.28		17	.11	
Didactic instruction	Yes	85	.22	.07	3	− .00	− .21	26	.19	− .13	6	.13	.00	25	.28	.23	23	.08	.02
	No	43	.15		7	.21		6	.32		12	.13		8	.05		11	.07	
Structural components																			
Parental involvement	Yes	6	.03	− .18	0	–	–	0	–	–	2	–	–	0	–	–	4	− .02	− .11
	No	122	.21		10			32			16			33			30	.09	
Whole school involved	Yes	17	.36	.19	0	–	–	4	.37	.16	2	–	–	9	.78	.67^**^	2	–	–
	No	107	.17		10			28	.22		16			24	.11		30		
Individual part	Yes	27	.10	− .14	0	–	–	8	.15	− .10	5	.14	.02	7	.07	− .20	7	.04	− .04
	No	101	.23		10			24	.25		13	.12		26	.27		27	.09	
Number of sessions	I		.21	.04		.13	.22		.23	− .01		.11	.04		.23	.11		.09	.04
Number of components	I		.19	− .01		.07	.16		.23	.00		.11	.04		.22	− .00		.08	.01

†*p* ≤ .10, *p ≤ .05, **p ≤ .01

There were also some relations between components and intervention effects showing a trend towards significance (*p* < 0.10). Increasing students’ self-efficacy was related to stronger intervention effects on resilience. Practicing skills during the intervention and having group discussions were related to stronger effects on self-regulation. Last, multimedia use was related to stronger effects on internalizing behavior.

#### Interpersonal Domain

Teaching students to regulate their emotions was significantly related to stronger intervention effects at follow-up on the interpersonal domain in general (see Table 3). Concerning the subdomains, increasing students’ self-efficacy, facilitating insight building, modeling desired behaviors, and setting goals were related to stronger intervention effects on sexual health.

**Table 3:** Effect sizes of interventions with and without components stimulating competencies and preventing problems in the interpersonal domain

Component		Interpersonal			Sexual health			Social competence			Aggression			Bullying		
		#ES	ES	*B*	#ES	ES	*B*	#ES	ES	*B*	#ES	ES	*B*	#ES	ES	*B*
Content components																
Emotion regulation	Yes	72	.23	.14*	8	.13	.05	18	.14	.08	30	.28	.20	8	.33	.31^†^
	No	83	.09		38	.09		4	.06		28	.08		13	.02	
Assertiveness	Yes	48	.10	− .08	17	.15	.11^†^	14	.05	− .11^†^	13	.05	− .21	4	− .03	− .19
	No	107	.18		29	.04		8	.16		45	.25		17	.16	
Self-efficacy	Yes	24	.21	.07	7	.26	.20^**^	9	.11	− .00	4	.31	.14	4	.21	.09
	No	131	.14		39	.06		13	.11		54	.18		17	.12	
Self-control	Yes	25	.13	− .03	5	.19	.11	0	–	–	20	.10	− .10	0	–	–
	No	130	.16		41	.08		22			38	.20		21		
Insight building	Yes	57	.19	.05	7	.26	.20^**^	9	.13	.03	30	.13	− .09	4	.06	− .11
	No	98	.14		39	.06		13	.10		28	.22		17	.17	
Cognitive coping	Yes	52	.12	− .06	23	.03	− .09	9	.14	.04	11	.13	− .09	3	− .10	− .30
	No	103	.17		23	.12		13	.09		47	.22		18	.20	
Relaxation	Yes	15	.22	.07	5	.19	.11	3	.02	− .11	6	.34	.18	1	–	–
	No	140	.15		41	.08		19	.13		52	.16		20		
Social skills	Yes	74	.20	.11	9	.14	.06	19	.11	− .02	28	.23	.14	10	.23	.25
	No	81	.09		37	.08		3	.13		30	.09		11	− .01	
Problem solving	Yes	81	.16	− .00	29	.10	.00	9	.09	− .04	36	.19	.00	7	.12	− .03
	No	74	.16		17	.10		13	.13		22	.19		14	.15	
Peer resistance	Yes	25	.14	− .02	1	–	–	2	–	–	22	.18	− .01	0	−	–
	No	130	.16		45			20			36	.19		21		
Instructional components																
Practice	Yes	103	.18	.06	17	.10	− .01	16	.13	.05	53	.20	.05	9	.22	.11
	No	52	.12		29	.11		6	.08		5	.15		12	.11	
Modeling	Yes	39	.17	.01	11	.18	.13*	11	.13	.03	14	.15	− .06	2	–	–
	No	116	.15		35	.05		11	.10		44	.21		19		
Discussion	Yes	99	.15	− .02	21	.10	− .01	16	.06	− .12^†^	47	.18	− .02	8	.22	.11
	No	56	.17		25	.10		6	.18		11	.20		13	.11	
Goal setting	Yes	22	.27	.14	7	.26	.20^**^	5	.14	.04	5	.33	.16	0	–	–
	No	133	.13		39	.06		17	.11		53	.17		21		
(Self-)monitoring	Yes	14	.13	− .03	0	–	–	5	.02	− .11	4	.11	− .09	0	–	–
	No	141	.16		46			17	.13		54	.20		21		
Multimedia	Yes	66	.17	.03	17	.13	.09	5	.10	− .02	33	.20	.01	4	.21	.09
	No	89	.15		29	.04		17	.12		25	.18		17	.12	
Homework	Yes	17	.29	.15	0	–	–	4	.24	.15	11	.26	.10	1	–	–
	No	138	.14		46			18	.09		47	.16		20		
Didactic instruction	Yes	98	.17	.04	43	.10	.03	11	.16	.11	23	.20	.02	15	.17	.17
	No	57	.13		3	.07		11	.05		35	.18		6	.01	
Structural components																
Parental involvement	Yes	66	.07	− .12	19	.03	− .09	5	.08	− .04	31	.10	− .15	11	− .01	− .30^†^
	No	89	.19		27	.12		17	13		27	.25		10	.29	
Whole school involved	Yes	54	.17	.00	27	.08	− .04	5	.08	− .04	7	.25	.05	10	.16	.05
	No	99	.17		19	.12		17	.13		49	.20		11	.11	
Individual part	Yes	22	.04	− .15^†^	3	.07	− .03	5	.06	− .07	3	− .04	− .28	11	.02	− .28
	No	133	.19		43	.10		17	.13		55	.23		10	.30	
Number of sessions	I		.17	.02		.09	− .03		.10	.02		.23	− .00		.23	.08
Number of components	I		.16	.03		.10	.03		.12	− .02		.19	− .00		.16	.03

†*p* ≤ .10, *p ≤ .05, **p ≤ .01

Again, some associations between components and intervention effects showed a trend towards significance (*p* < 0.10). The inclusion of an individual part in the intervention was related to weaker intervention effects on the interpersonal domain in general. Regarding the subdomains, teaching assertiveness was related to stronger intervention effects on sexual health. In contrast, teaching assertiveness and having group discussions were related to weaker effects on social competence. Teaching students to regulate their emotions was related to stronger intervention effects on bullying, whereas involving parents in the intervention was related to weaker effects on this outcome.

## Discussion

Although universal secondary school-based interventions aim to stimulate students’ long-term development, intervention effects generally seem to fade-out over time (e.g., Dray et al., 2017; Taylor et al., 2017). More insight is needed into which intervention effects last long-term and which components may generate or maintain these long-term effects. Our meta-analysis focused on a broad range of competencies and problems in which we made a distinction between outcomes in the intrapersonal domain and outcomes in the interpersonal domain. We explicitly examined interventions targeting adolescents. Given that long-term intervention effects seem especially small for this group, it is important to gain knowledge about interventions’ long-lasting impact on their psychosocial development and which intervention components are associated with stronger effects at follow-up for adolescents specifically.

### Long-term Effects of Universal School-Based Interventions

The intervention effects at follow-up were positive, albeit small, on multiple competencies and problems. More specifically, in the intrapersonal domain we found small positive effects on self-esteem, self-regulation, general wellbeing, and internalizing problems, but found no significant effects on resilience. In the interpersonal domain, we found small positive effects on sexual health, social competence, and aggression, though no significant effects on bullying. The effects were generally unaffected by the duration of the follow-up period. Only intervention effects on general wellbeing and social competence seemed to be affected by the duration of follow-up. Regarding general wellbeing, intervention effects became stronger over time. Accordingly, intervention effects at post-measurement on general wellbeing found in the meta-analysis of Mertens et al. (2020) were smaller than the intervention effects at follow-up on general wellbeing found in the current meta-analysis, respectively *d* = 0.13 and *d* = 0.22. This increase in effect indicates that it might take some time before students can benefit optimally from intervention content related to their general wellbeing. Students may need time to practice skills learned during the intervention and gain confidence in using these skills (Van Aar et al., 2017). Over time, they may apply these skills to deal effectively with situations encountered after the intervention, strengthening their sense of agency and improving their general wellbeing (Bell et al., 2013).

Concerning social competence, intervention effects became weaker over time. Although minimal, this decrease in intervention effects over time also shows when comparing the effect at post-measurement found by Mertens et al. (2020) with the effect at follow-up found in the current meta-analysis, respectively *d* = 0.16 and *d* = 0.11. Newly learned social skills may especially need to be practiced on a regular basis in order to be maintained. Based on these findings, practitioners may consider implementing booster sessions after the intervention to sustain intervention effects over time on social competence (Van Aar et al., 2017). However, before booster sessions are implemented, more research is needed to inform the development of effective booster sessions, as booster sessions are not inherently related to stronger intervention effects over time (Lochman et al., 2014; Neil & Christensen, 2009).

### Intervention Components and Follow-Up Effects

Components seemed to be related to effect sizes on specific outcomes rather than across several outcomes within a domain. In fact, our results suggest that some components may be associated with stronger effects on one outcome and weaker effects on another outcome. For example, having group discussions during the intervention was related to stronger effects at follow-up on self-regulation and internalizing behaviors. However, it was also related to weaker effects at follow-up on social competence. Moreover, components related to stronger intervention effects at follow-up were not necessarily frequently implemented. For instance, setting goals was related to stronger intervention effects, though this component was only implemented in 15% and 19% of the interventions targeting the intra- and interpersonal domains, respectively. These findings emphasize the importance of carefully selecting components that match the competency or problem the intervention addresses and that have an evidence base for their effectiveness.

Content components seemed particularly important to stimulate follow-up effects on competencies and problems in the interpersonal domain. Teaching students social-emotional skills, such as emotion regulation, self-efficacy, and insight building, was related to stronger intervention effects at follow-up. Interestingly, in other studies, focusing on social-emotional skills was related to negative effects immediately after the intervention. In the meta-analysis of Gaffney et al. (2021), which examined anti-bullying interventions, teaching students social-emotional skills during the intervention was related to weaker effects immediately after the intervention on both bullying and victimization. Similarly, the meta-analysis of Mertens et al. (2020), which examined universal school-based interventions, found that teaching students to regulate their emotions, a specific social-emotional skill, was related to weaker effects at post-measurement on self-esteem and bullying. These contrasting results may be explained within the framework of the Healthy Context Paradox (Salmivalli, 2018), which states that individuals who experience problems in a positive context may develop more problems because they feel they cannot benefit from the positive context, while others can. Conceivably, during interventions that teach social-emotional skills, students might become more aware of the discrepancy between their daily life experiences and societal norms. Due to this awareness, they might report more problems immediately after the intervention. At the same time, the intervention can provide students the skills needed to address and overcome these problems, resulting in more competence and fewer problems over time. Based on this process, negligible to negative intervention effects would be expected at post-measurement, and positive effects would be expected at follow-up. This delayed benefit from the intervention can be regarded as a sleeper effect of components in which the intervention’s content may require additional time to settle in (Bell et al., 2013).

Instructional components appeared particularly relevant for fostering follow-up effects on competencies and problems in the intrapersonal domain. In line with previous meta-analyses examining short-term effects (e.g., Mertens et al., 2020; Sheridan et al., 2019), active learning approaches, such as practicing skills, modeling desired behaviors, setting goals, and using multimedia, were related to stronger intervention effects at follow-up. The active learning approach through group discussions during the intervention is an ambiguous component. While this component was related to stronger effects on self-regulation and internalizing behavior, it was related to weaker effects on social competence. Implementing successful group discussions requires well-developed social and communication skills from facilitators (Wong et al., 2019). Perhaps a certain level of social competence is also required for students to participate successfully in discussions when implemented in the school context. Hence, using active learning approaches appears a promising intervention method, though it may be important to match the learning approach with the competencies and strengths of the participating students and facilitators.

Structural components were related to only a few follow-up intervention effects, suggesting that this type of component may be less relevant for establishing long-term intervention effects. In other words, to establish long-term intervention effects, what students learn (i.e., content components) and how they learn it (i.e., instructional components) may be more important than the way the intervention is set up (i.e., structural components). Although a whole school approach was related to stronger effects on students’ general wellbeing, components indicative of more extensive interventions were not consistently related to stronger intervention effects. Interventions that involved parents were related to weaker effects on bullying, and those that included an individual aspect were related to weaker effects on the interpersonal domain in general. These findings suggest that investing in longer and more extensive school-based intervention may establish stronger effects in the short-term (e.g., Gaffney et al., 2021; Mertens et al., 2020; Sheridan et al., 2019), but they may be less worthwhile in the long run.

## Limitations

The current meta-analysis has several limitations. First, the associations between components and intervention effects are based on correlational data and should therefore be regarded as hypotheses generating results, indicating which components are particularly interesting to examine further. To stimulate the formulation of hypotheses, we reported significant findings as well as findings with a trend towards significance. Relatedly, we analyzed components that were embedded within intervention programs that included multiple components. As a result, we cannot draw conclusions about the effectiveness of specific components on their own. Second, to code the components, we were dependent on the sufficiency of the intervention descriptions. Although we searched for additional information regarding the interventions in appendices, other articles and on websites, if a component was not mentioned in those descriptions, we coded it as absent. Hence, some components may falsely be coded as absent. Vice versa, components described as part of the intervention may not have been implemented, even if they were coded as such. Third, due to the low implementation frequency of some components, some analyses regarding the components had relatively low power or could not be conducted. To avoid marking components prematurely as irrelevant due to low power, we interpreted associations between components and long-term effects that were significant and that showed a trend towards significance. This approach optimizes the relevance of our results for future research (i.e., hypotheses generation).

## Conclusion

Taken together, our meta-analysis has two important implications. First, universal secondary school-based interventions show small positive long-term effects on multiple outcomes. While they are small, these effects show that interventions can have a long-lasting effect and have the potential to cultivate important aspects of adolescents’ psychosocial development. On the other hand, the small effect sizes indicate that there is room for improvement. To increase our understanding of long-term intervention effects, intervention studies should conduct follow-up assessments to examine long-term effectiveness and the relationships between long-term effects and the intervention’s components. Unfortunately, conducting a follow-up is not yet the standard in intervention research.

Second, components seem to be related to intervention effects on specific outcomes, sometimes with opposing effects. This raises the question of whether the heterogeneity in problems that exists in the total student population can be addressed by implementing a single intervention with a broad aim. Some components appear related to stronger long-term intervention effects on one outcome, though they are related to weaker effects on another outcome. In addition, some components appear associated with stronger short-term effects, though they show weaker long-term effects (or vice versa) on specific outcomes (Mertens et al., 2020). Gaining insight into these conflicting effects is critical. Thus, future research should examine the extent to which components relate to effects on different outcomes and how components can be combined into one intervention that addresses a broad range of outcomes. Such interventions should include components related to stronger effects on both the short-term and the long-term.

The effectiveness of an intervention is not only determined by the implemented components, but also by numerous other factors (e.g., the training, enthusiasm and characteristics of facilitators, the fit between the intervention content and the class- and school climate, and the resources and time available to invest in implementation). However, a better understanding of long-term intervention effects and the intervention components associated with such effects can facilitate the development of optimized interventions that exhibit increased long-term effectiveness.

## Supplementary Information

Below is the link to the electronic supplementary material.

Supplementary file1 (DOCX 26 kb)

## Data Availability

Not applicable.
