# Supplementary material for: Components Related to Long-Term Effects in the Intra- and Interpersonal Domains: A Meta-Analysis of Universal School-Based Interventions
Source: Clin Child Fam Psychol Rev. 2022 Jul 31;25(4):627–45. doi: 10.1007/s10567-022-00406-3 (PMC9622567; doi:10.1007/s10567-022-00406-3)
Supplement: Supplementary file 1 — Supplementary file1 (DOCX 26 kb) [file 10567_2022_406_MOESM1_ESM.docx]

Appendix A – Definitions of the general domains and the subdomains

**Intrapersonal domain** = the ability to manage one’s own feelings, emotions, and attitudes about the self (Barber, 2005). This domain concerns the subjective processing of behaviors, thoughts, and emotions pertained by the individual self (Dufner et al., 2019; Finkel & Vohs, 2006). Evaluating and regulating one’s own inner world and experiences can facilitate positive personal functioning (e.g., psychological wellbeing and resilience), whereas difficulties in this process can increase the chance of developing psychological problems (e.g., internalizing behavior; Dufner et al., 2019).

- **Resilience** = the ability to bounce back from the variety of challenges that can arise in life (Campbell-Sills & Stein, 2007).
- **Self-esteem** = the extent to which individuals like themselves as a person in general or on specific domains (Wichstraum, 1995).
- **Self-regulation** = the automatic or deliberate modulation of affect, behavior, and cognition (Karoly, 1993).
- **General wellbeing** = the presence of positive emotions and life satisfaction, and the absence of negative feelings (Robitail et al., 2007).
- **Internalizing problems** = a broad range of mood and anxiety behaviors directed towards one’s inner world putting an individual at risk of developing later mood and anxiety disorders (Petty et al., 2008).

**Interpersonal domain** = the ability to build and maintain positive relationships with others, to understand social situations, roles and norms, and to respond appropriately (Pellegrino & Hilton, 2012; Shek & Leung, 2016). By planning one’s own behavior and predicting the behavior of others one can act in a socially appealing way, such as building positive interpersonal relations, or in a more destructive way, such as behaving aggressively or bullying (Finkel & Vohs, 2006).

- **Sexual health** = one’s coping skills and attitudes in romantic situations and relations (De Graaf et al., 2005).
- **Social competence** = skills to successfully and positively interact with others in social situations (Shek & Leung, 2016).
- **Aggression** = intentional, proactive or reactive, behavior intended to hurt. Others (Coyne et al., 2010).
- **Bullying** = repeated, over time, exposure to negative actions (physical, verbal, or nonverbal) with the intention to hurt or bring discomfort upon another by one or more other individuals who are stronger (i.e., imbalance in strength; Olweus, 1993).

**References**

Barber, B. K. (2005). Positive interpersonal and intrapersonal functioning: An assessment of measures among adolescents. In K. A. Moore, & L. H. Lippman (Eds.), *What do children need to flourish: Conceptualizing and measuring indicators of positive development* (pp. 147–161). New York, NY: Springer Science and Business Media.

Campbell-Sills, L., & Stein, M. B. (2007). Psychometric analysis and refinement of the Connor-Davidson Resilience Scale (CD-RISC): Validation of a 10-item measure of resilience. *Journal of Traumatic Stress, 20(6),* 1019–1028. <https://doi.org/10.1002/jts.20271>

Coyne, S. M., Nelson, D. A., & Underwood, M. (2010). Aggression in children. In P. K. Smith, & C. H. Hart (Eds.). *The Wiley-Blackwell handbook of childhood social development* (pp. 491–509). Hoboken, New Jersey: Wiley.

De Graaf, H., Meijer, S., Poelman, J., & Vanwesenbeeck, I. (2005). *Seks onder je 25^e^* [Sex under the age of 25]. Delft: Uitgeverij Eburon.

Dufner, M., Gebauer, J. E., Sedikides, C., & Denissen, J. J. A. (2019). Self-enhancement and psychological adjustment: A meta-analytic review. *Personality and Social Psychology Review, 23,* 48–72. <https://doi.org/10.1177/1088868318756467>

Finkel, E. J., & Vohs, K. D. (2006). Introduction. Self and Relationships. In K. D. Vohs, & E. J. Finkel (Eds.), *Self and relationships: Connecting intrapersonal and interpersonal processes* (pp. 1–9)*.* New York, NY: The Guilford Press.

Karoly, P. (1993). Mechanisms of self-regulation: A systems view. *Annual Review of Psychology, 44,* 23–52.

Olweus, D. (1993). *Bullying at school: What we know and what we can do.* Oxford: Blackwell Publishers.

Pellegrino, J. W., & Hilton, M. L. (2012). *Education for life and work: Developing transferable knowledge and skills in the 21^st^ century.* National Academy of Sciences, Washington, DC.

Petty, C. R., Rosenbaum, J. R., Hirshfeld-Becker, D. R., Henin, A., Hubley, S., LaCasse, S., Faraone, S., & Biederman, J. (2008). The Child Behavior Checklist broad-band scales predict subsequent psychopathology: A five-year follow-up. *Journal of Anxiety Disorders, 22,* 532–539.

Robitail, S., Ravens-Sieberer, U. R., Simeoni, M., Rajmil, L., Bruil, J., Power, M., Duer, W., Cloetta, B., Czemy, L., Mazur, J., Czimbalmos, A., Tountas, Y., Hagquist, C., Kilroe, J, Auquire, P., & The KIDSCREEN Group. (2007). Testing the structural and cross-cultural validity of the KIDSCREEN-27 quality of life questionnaire. *Quality of Life Research, 16,* 1335–1345. <https://doi.org/10.1007/s11136-007-9241-1>

Shek, D. T. L., & Leung, J. T. Y. (2016). Developing social competence in a subject on leadership and intrapersonal development. *International Journal on Disability and Human Development, 51,* 165–173. <https://doi.org/10.1515/ijdhd-2016-0706>

Wichstraum, L., (1995). Harter’s Self-Perception Profile for adolescents: Reliability, validity, and evaluation of the question format. *Journal of Personality Assessment, 65,* 100–116.

Appendix B – complete list of search terms

| Category | Term |
| --- | --- |
| Program descriptors | School, schools, class, classroom, classes, school-based, “school based”, group, group-based, “group based”, high-school, “secondary school” |
| Evaluation descriptors | Intervention, preventive, prevention, program* |
| Program targets | Adolescent, adolescence, adolescents, youth, teen, teenager, teenagers |
| Program outcomes | “Psychological well-being”, resilience, resiliency, “emotional adjustment”, “self-efficacy”, “social isolation”, “social identification”, psychosexual, communication, communicative, anger, aggression, aggressive, “social support”, “social safety”, “psychosocial well-being”, “emotion regulation”, “self-regulation”, “self-control”, “self-esteem”, “self-worth”, “self-confidence”, “peer problems”, “relational aggression”, “externalizing behavior”, “externalising behaviour”, “internalizing behavior”, “internalising behaviour”, violen*, “social competence”, “positive social behavio*”, “emotional distress”, “prosocial behavio*” |
| Restrictions | Substance, drug, drugs, alcohol, smoking, lifestyle, “weight loss”, diet, dietary, obesity, obese, disorder*, disease, clinical, patient, patients, HIV, aids, illness, disabilit*, disabled, suicide, suicidal, anorexia, eating, pregnan*, nurs*, neural, neuron, college, university, “primary school”, preschool |

Appendix C – Definitions of Components

| **Content components** *= specific skills adolescents learn to promote positive outcomes* | | |
| --- | --- | --- |
|  | Emotion regulation | Strategies to help youth identify and appropriately express emotions (including aggression) |
|  | Assertiveness | Exercises designed to promote the youth’s ability to assert his or her needs appropriately with others |
|  | Self-efficacy | Techniques and training to enhance self-confidence and improve self-efficacy |
|  | Self-control | Strategies to help youth interrupt undesired behavioral tendencies (e.g., impulses) and refrain from acting on them. |
|  | Insight building | Activities specifically designed to help a youth achieve greater self-understanding and adjust attitudes |
|  | Social skills | Training youth how to communicate more effectively with others and providing constructive information, training, and feedback to improve interpersonal verbal or non-verbal functioning |
|  | Problem solving | Training in the use of techniques, discussions, or activities designed to bring about solutions to social, emotional, or behavioral problems |
|  | Peer resistance | Techniques or training to learn youth how to resist pressure from peers |
| **Instructional components** = *techniques and methods of information delivery used by the intervention facilitator* | | |
|  | Practice | Practicing of a desired behavior during session (e.g., role-play) |
|  | Modeling | Demonstration to the youth of a desired behavior |
|  | Discussion | Discussion of topics within a group |
|  | Goal setting | The explicit selection of a therapeutic goal for the purpose of working toward achieving that goal |
|  | (Self-)monitoring | The repeated measurement of a target index (by the youth) |
|  | Relaxation | Techniques or exercises designed to induce physiological calming |
|  | Multimedia | The use of multimedia to bring or reinforce new knowledge or skills |
|  | Cognitive coping | Any techniques designed to alter interpretation of events or deal with stressful situations through examination of the youth’s reported thoughts (e.g., cognitive restructuring) |
|  | Homework | Written, verbal, or behavioral assignments to complete between sessions |
|  | Didactic instruction | The formal (usually didactic) review of information (e.g., psychoeducation) |
| **Structural components** = *describe the structure of the intervention that might impact results* | | |
|  | Parental involvement | Parents are directly or indirectly involved during the intervention |
|  | Whole school involved | The school staff is directly or indirectly involved during the intervention |
|  | Individual part | The intervention includes additional individual guidance or explicit individual progress through the intervention (e.g., expressive writing, internet-based intervention) |
|  | Number of sessions | Number of sessions of the intervention |
|  | Number of components | Number of components implemented in the intervention |

*Note.* We reviewed the components of the reviews and meta-analyses of Kaminski and colleagues (2008), Onrust and colleagues (2016), Peters and colleagues (2009), and Van der Put and colleagues (2018). Based on this broad theoretical basis, we adjusted some components of Boustani and colleagues (2015) due to highly overlapping content and co-occurrence (i.e., we combined *communication skills* and *social skills*; we combined *cognitive coping* and *coping skills*; *emotion regulation* contains *anger management*; *practice* contains *role-play*) and we deleted some components due to low frequency (i.e., civic responsibility, support networking).

Appendix D – Descriptives of Included Publications

| Reference | Name of the intervention | Target | Outcomes | Grades |
| --- | --- | --- | --- | --- |
| Allen 2021 | The Connection Project | Intra and inter | Social competence, internalizing | NR |
| Baker 2014 | Respect | Inter | Sexual health | 9-12 |
| Bonell 2017 | Learning Together intervention | Inter | Wellbeing, aggression, bullying | 7 |
| Bull 2009 | The fairplayer.manual | Inter | Bullying | 9-11 |
| Burckhardt 2017 | Acceptance and Commitment Therapy (ACT) | Intra | Internalizing, wellbeing | 10 |
| Burckhardt 2018 | Dialectical behavior therapy skills group | Intra | Self-regulation, internalizing, aggression | 10 |
| Calear 2009 | MoodGym | Intra | Internalizing | 8-10 |
| Calvete 2019 | Incremental theory of personality intervention | Inter | Bullying | 8-10 |
| Caprara 2014 | CEPIDEA | Inter | Self-esteem, aggression, social competence | 7 |
| Carissoli 2019 | EmotivaMente | Intra and inter | Social competence, self-regulation | 9 |
| Castillo 2013, Ruiz-Aranda 2012 | INTEMO | Intra and inter | Aggression, self-regulation, self-esteem, internalizing, wellbeing, social competence | 7 |
| Challen 2014 | UK Resilience Program | Intra | Internalizing, social competence | 6 |
| Coelho 2015 | Positive Attitudes | Intra and inter | Self-esteem, self-regulation, internalizing, social competence | 7-9 |
| Corder 2020 | GoActive | Inter | Self-esteem, wellbeing | 8 |
| Cross 2016 | Cyber Friendly School | Inter | Bullying | 8, 9 |
| De Graaf 2016 | Rock and Water | Inter | Self-esteem, self-regulation, sexual health | 9, 10 |
| De Villiers 2012 | Resilience program | Intra | Resilience, self-esteem, self-regulation, social competence | 6 |
| Edwards 2019 | Bring in the Bystander – High School Curriculum | Intra | Romantic relations | 9-12 |
| Foshee 2005 | Safe Dates | Inter | Aggression | 8, 9 |
| Ghobari Bonab 2021 | Forgiveness education program | Inter | Aggression, social competence | 8 |
| Gollwitzer 2007 | Vienna Social Competence Training (ViSC) | Inter | Aggression | 6-8 |
| Horn 2010 | JES! Jugendpräventionsprogramm mit Expressivem Schreiben | Intra | Wellbeing | 8 |
| Jaycox 2006 | Ending violence: A curriculum for educating teens on domestic violence and the las | Inter | Sexual health | 9 |
| Jewkes 2019 | Skhokho intervention | Intra and inter | Aggression, internalizing, bullying | 8 |
| Jewkes 2019 | Skhokho intervention and family workshop | Intra and inter | Aggression, internalizing, bullying | 8 |
| Kaveh 2014 | Peer led training program | Intra | Self-esteem | 7 |
| Kiselica 1994 | Stress inoculation training with assertiveness training | Intra | Internalizing, wellbeing | 9 |
| Kozina 2018a  Kozina 2018b | My FRIENDS | Intra | Internalizing, aggression | 8 |
| Lamke 1988 | Cognitive-behavior modification program | Intra | Self-esteem | 9 |
| Meyer 2004 | Get real about violence | Inter | Aggression | 7 |
| Muck 2018 | Scientist practitioner program | Inter | Sexual health | 8, 9 |
| Orpinas 1995 | Second Step: A violence prevention curriculum | Inter | Self-esteem, aggression | 6 |
| Pannebakker 2019 | Skills 4 Life | Intra and inter | Social competence, self-esteem, internalizing, wellbeing | 7-9 |
| Perkins 2021 | An enhanced psychological mindset session for adolescents | Intra | Self-esteem, internalizing, wellbeing | 11-12 |
| Peskin 2019 | Me & You: Building Healthy Relationships | Intra and inter | Sexual health, social competence, self-regulation | 6 |
| Richardson 2009 | BodyThink | Intra | Self-esteem, bullying | 7 |
| Ruini 2009, Tomba 2010 | Well-being Therapy (WBT) with added cognitive-behavioral packages | Intra | Self-regulation, internalizing, wellbeing, aggression, social competence, resilience, self-esteem | 6, 9, 10 |
| Sharma 2020 | Setu | Inter | Aggression, bullying | 8 |
| Shoshani 2014, Shoshani 2016 | Maytiv School Program | Intra and inter | Self-esteem, wellbeing | 7-9 |
| Simons-Morton 2005 | Going Places Program | Inter | Aggression | 6 |
| Singh 2019 | Resilient Families | Intra | Self-regulation, internalizing | 7 |
| Singh 2019 | Resilient Families including Parenting Adolescents: A creative experience | Intra | Self-regulation, internalizing | 7 |
| Soliday 2004 | Expressive writing Intervention | Intra | Internalizing, wellbeing | 8 |
| Solomontos-Kountouri 2016 | ViSC social competence program (with added parental component) | Inter | Aggression, bullying | 7, 8 |
| Stevens 2000 | Flemish anti-bullying program | Inter | Social competence, bullying | NR |
| Sundgot-Borgen 2019, Sundgot-Borgen 2020 | Healthy Body Image Intervention | Intra | Self-esteem, wellbeing | 10 |
| Thomaes 2009 | Self-affirmation intervention | Inter | Self-esteem, aggression | 7, 8 |
| Tomba 2010 | Anxiety management intervention | Inter and intra | Aggression, social competence, resilience, self-esteem, self-regulation, internalizing, wellbeing | 6 |
| Volanen 2020 | .b (Stop and Breathe/Be) | Intra | Resilience, internalizing, wellbeing | 6-8 |
| Volanen 2020 | Relax | Intra | Resilience, internalizing, wellbeing | 6-8 |
| Williams 2015, Miller 2015 | Start strong: Building healthy teen relationships | Inter | Aggression, bullying, sexual health, social competence | 7 |
| Wolfe 2009 | Fourth R: Skills for youth relationships | Inter | Aggression, sexual health | 9 |

*Note.* Intra = Intrapersonal domain; Inter = Interpersonal domain; NR = Not reported
